# Leveraging Large Language Models to Extract Prognostic Pathology Features in Ewing Sarcoma

**DOI:** 10.64898/2026.02.20.707103

**Published:** 2026-02-21

**Authors:** Jingwei Huang, Ayesha Batool, Zifan Gu, Zhuo Zhao, Bo Yao, Jennifer Black, Jessica Davis, Alyaa al-Ibraheemi, Steven DuBois, Don Barkauskas, Subhash Ramakrishnan, David Hall, Patrick Grohar, Yang Xie, Guanghua Xiao, Patrick J. Leavey

**Affiliations:** 1 O’Donnell School of Public Health, UT Southwestern Medical Center Dallas; 2 Children’s Health / Children’s Medical Center Dallas; 3 Department of Pediatrics, UT Southwestern Medical Center Dallas; 4 Children’s Hospital Colorado; 5 Riley Hospital for Children; 6 Dana-Farber / Boston Children’s Cancer and Blood Disorders Center and Harvard Medical School; 7 Children’s Oncology Group; 8 CS Mott Children’s Hospital

## Abstract

**Background:**

Current risk stratification for Ewing sarcoma relies heavily on clinical factors such as metastatic status, failing to capture histologic heterogeneity as a potential prognostic indicator. Although pathology reports contain rich biological data, this information remains locked in unstructured narrative text, limiting large-scale retrospective analyses. We aimed to validate the utility of Large Language Models (LLMs) for scalable data abstraction and to identify prognostic histologic features from a large multi-institutional cohort.

**Methods:**

We conducted a retrospective cohort study using data from six Children’s Oncology Group (COG) clinical trials. We utilized an LLM-based pipeline (OpenAI o3) to extract structured variables—including immunohistochemical (IHC) markers and CD99 staining patterns—from digitized, Optical Character Recognition (OCR)-processed pathology reports. Extraction accuracy was validated against a human-annotated ground truth (n=200) and cross-validated against senior experts (n=48). We assessed the association between extracted features and Overall Survival (OS) using Kaplan-Meier analysis and multivariable Cox proportional hazards regression, adjusting for metastatic status.

**Findings:**

We analyzed 931 diagnostic pathology reports spanning over 19-years. The LLM achieved a weighted average accuracy of 94% across 17 IHC markers; in a cross-validation subset, the LLM outperformed human annotators (weighted average accuracy over 15 IHC markers: LLM o3: 98.1%, a resident specialist 91.4%, and a senior expert 95.9%). Survival analysis identified Neuron-Specific Enolase (NSE) and S100 as significant prognostic biomarkers. After adjusting for metastatic status, NSE positivity was associated with significantly inferior survival (HR 2.15, 95% CI 1.15–4.02, p=0.016); this risk was most pronounced in patients with non-metastatic disease (HR 5.64, p=0.0055). Conversely, S100 positivity was associated with improved survival (HR 0.58, 95% CI 0.34–1.00, p=0.046).

**Interpretation:**

LLM-assisted extraction of pathology variables is highly accurate and scalable, capable of unlocking “dark data“ from historical clinical trials. We identified NSE as a potent risk factor and S100 as a protective marker in Ewing sarcoma, particularly in localized disease. These findings suggest that AI-derived histologic data can refine risk stratification and, if validated, warrant inclusion in future prospective trials.

## Introduction

Current risk stratification for Ewing sarcoma largely relies on clinical factors, such as the presence of metastatic disease and primary tumor size. While these factors are robust, they fail to capture histologic heterogeneity, which may serve as a critical but underutilized prognostic indicator. Although pathology reports and histology are universally available at the time of diagnosis, extracting structured data from narrative documents is labor-intensive and has historically limited large-scale retrospective analyses. Consequently, vast amounts of potentially practice-changing data remain locked in unstructured free text, inaccessible to conventional statistical analysis.

Recent advances in artificial intelligence, specifically Large Language Models (LLMs)^1–8^, offer a transformative solution to this data bottleneck. Trained on massive corpora of text data spanning diverse domains of human knowledge, LLMs possess the unique capability to analyze complex, unstructured medical literature and clinical notes. By applying these models to extract structured variables from narrative reports^9–12^, healthcare researchers can unlock the “dark data” of historical clinical trials. This process is crucial for identifying patterns, trends, and correlations that might otherwise remain hidden due to the prohibitive time and cost of manual data abstraction.

In this study, we sought to validate the utility of LLMs for scalable pathology data abstraction within a high-value clinical cohort. We assembled a large dataset of patients with Ewing sarcoma enrolled in six distinct Children’s Oncology Group (COG) clinical trials over a 19-year period. Our objective was to determine whether LLM-based extraction of variables from digitized pathology reports could accurately identify histologic biomarkers associated with survival. Specifically, we investigated the prognostic significance of immunohistochemical markers, including Neuron-Specific Enolase (NSE) and S100, thereby demonstrating that automated extraction pipelines can reveal novel biological insights from historical data to inform future risk stratification^13–15^.

## Materials and Methods

### Study Design and Data Sources.

**Figure 1.**
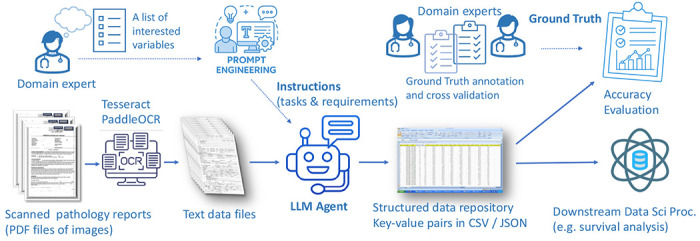
Overall workflow

The overall workflow of this research is illustrated in Figure 1. We conducted a retrospective cohort study utilizing data from patients enrolled in six distinct Children’s Oncology Group (COG) clinical trials for Ewing sarcoma: AEWS0031, AEWS1031, AEWS1221, AEWS02B1, APEC14B1 and AEWS07B1. Primary outcomes have been reported for each of the 3-interventional trials (AEWS0031, AEWS1221, AEWS1031)^16–18^ while the other 3-trials were complementary biology trials. We secured 933 digitized diagnostic pathology reports corresponding to 931 unique patients; two patients had duplicate reports derived from different scans. In addition to the pathology documents, the COG Statistical Data Center provided a clinical data sheet containing structured baseline characteristics for 931 patients, including demographic details and metastatic status at diagnosis. A comprehensive summary of the patient cohort is provided in Table 1.

### Data Pre-processing and Optical Character Recognition.

Pathology reports were generated at the time of diagnostic biopsy for patients enrolled in COG clinical trials open at hundreds of different institutions across North America, spanning a collection period of 19 years. Submission of source documents as scanned Portable Document Format (PDF) images were required for clinical trial enrollment and, given the time span, were of varying clarity, language (English and French), and formatting. To convert these images into machine-readable text, we utilized Tesseract, an open-source Optical Character Recognition (OCR) engine. Given the historical nature of the documents, the raw OCR output contained significant noise, including typographical errors, non-English characters, artifactual symbols from scanning lines, and formatting disruptions. This unstructured and noisy text served as the primary input for the downstream Large Language Model (LLM) extraction pipeline.

### LLM-Based Feature Extraction.

We employed OpenAI’s o3 model to extract specific pathological features of interest from the OCR text. We developed a schema to extract two categories of histologic data: immunohistochemical (IHC) staining status and CD99 immunostaining patterns. For IHC analysis, we targeted 17 specific markers, including CD99 (MIC2), NSE, S100, NKX2.2, FLI-1, ERG, Myogenin, MyoD1, DUX4, WT1, ETV4, BCOR, LCA (CD45), Synaptophysin, Chromogranin, Desmin, and Vimentin. The LLM was prompted to classify each marker into one of three standardized values: “Positive,” “Negative,” or “Not Specified.” The prompt template and the JSON output data structure used for the LLM are provided in Supplementary Figure S1 and S2.

In addition to marker status, we utilized the LLM to characterize the spatial patterns of CD99 staining. Based on descriptive features in the text, the model categorized staining into specific patterns, including membranous versus cytoplasmic distribution, and diffuse versus patchy or focal intensity. The specific prompts and output templates for these tasks are detailed in Supplementary Figure S3.

### Ground Truth Validation and Performance Metrics.

To rigorously evaluate the accuracy of the LLM extraction, we established a human-annotated ground truth (GT) dataset. We randomly selected 200 reports from the total cohort of 933 scans. Three reports were subsequently excluded due to duplication or re-classification as non-Ewing sarcoma cases, resulting in a final validation set of 197 reports. Ewing sarcoma clinical specialists (a pediatric resident and a pediatric oncologist) manually reviewed the original PDF images to annotate the features, serving as the primary ground truth.

To assess inter-rater reliability and further validate the ground truth, all 197 images were annotated by the pediatric resident while a subset of 48 randomly selected cases underwent independent blinded review by the pediatric oncologist. Discrepancies between the annotators were resolved via a consensus panel discussion to establish the final ground truth. We then compared the performance of the LLM against both the Ewing sarcoma specialists individually and against the same consensus ground truth. Metrics calculated included accuracy, precision, recall, and F1-score. The comparative performance metrics are detailed in Table 3 and Table 4.

### Statistical Analysis.

The primary clinical endpoint was Overall Survival (OS), defined as the time from diagnosis to death or last known follow-up. Patients who were alive at the time of last contact were censored. Survival probabilities were estimated using the Kaplan-Meier method^15^, and differences between groups were assessed using the log-rank test. Life status was treated as a binary event indicator, and survival time was derived directly from the COG clinical dataset.

To evaluate the prognostic significance of the extracted features, we fit univariate and multivariable Cox proportional hazards regression models^14^. For the analysis of IHC variables, markers were treated as binary variables (Positive vs. Negative), with “Not Specified” values treated as missing data. Multivariable models included both the biomarker of interest and metastatic status at diagnosis to determine independence. We further performed subgroup analyses stratified by metastatic status (metastatic vs. non-metastatic) and tested for interactions between the biomarker and disease stage. All statistical analyses were performed using R statistical software. Survival curves were generated using the survival and survminer packages, with results summarized as Hazard Ratios (HR) and 95% Confidence Intervals (CI). Two-sided p-values < 0.05 were considered statistically significant.

## Results

### Immunohistochemical Marker Extraction and LLM Performance.

The LLM pipeline successfully processed pathology reports for 930 of the 931 unique patients, with only one exclusion due to an OpenAI API connection failure. We extracted data for 17 distinct immunohistochemical (IHC) markers; the full prevalence of these markers across the cohort is presented in Supplementary Table S1. As expected for Ewing sarcoma, CD99 (MIC2) was the most frequently reported positive marker (846 positive, 4 negative, 80 not specified). Other markers showed significant variability in reporting frequency and results, including desmin (8 positive, 576 negative, 346 not specified), NSE (95 positive, 70 negative, 765 not specified), and S100 (68 positive, 266 negative, 596 not specified).

**Figure 2.**
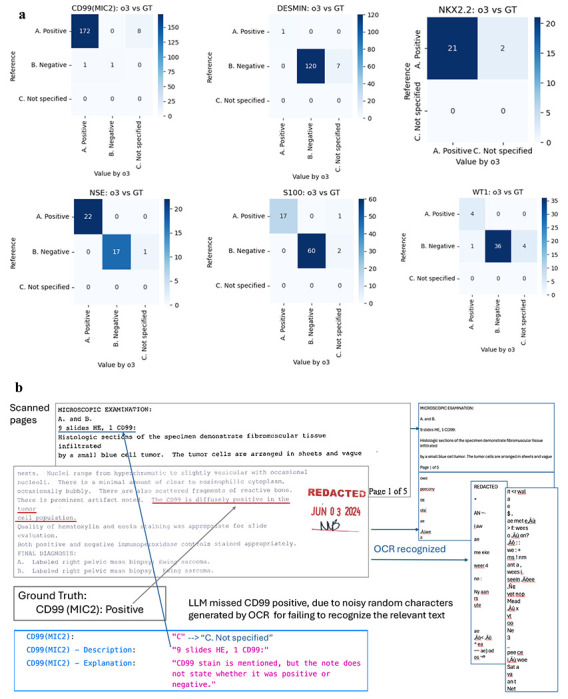
Representative confusion matrix in IHC data extraction

Despite the variable quality of the source documents, the LLM demonstrated high fidelity in data abstraction. Figure 2a displays the confusion matrices for six representative IHC markers, including CD99, NSE, S100, Desmin, NKX2.2, and WT1. The extraction performance was evaluated against the manually annotated testing dataset of 197 patients. As detailed in Table 2, the weighted average accuracy across all 17 IHC markers was 94%, with a weighted precision of 0.997 and an F1-score of 0.966. This high performance was achieved even in the presence of significant optical character recognition (OCR) noise, such as random character insertion and misaligned text, which is illustrated in Figure 2b.

To further benchmark the model’s capabilities, we conducted a rigorous cross-validation against human annotators on a subset of 48 randomly selected cases. BCOR and CCNB3 are not specified in all 48 cases, thus being excluded. Table 3 demonstrates that inter-human consistency between the two specialists was high, averaging 96.3% across markers. Using the consensus ground truth from a panel discussion of the team, Table 4 compares the accuracy of the human annotators against the LLM (o3 model). The pediatric resident achieved an average accuracy of 91.4%, while the pediatric oncologist achieved 95.9%. Notably, the LLM achieved an average accuracy of 98.1%, surpassing both human annotators. This suggests that the LLM is highly resilient to the fatigue and reading errors that can affect human review of poor-quality legacy documents.

### CD99 Staining Pattern.

**Figure 3.**
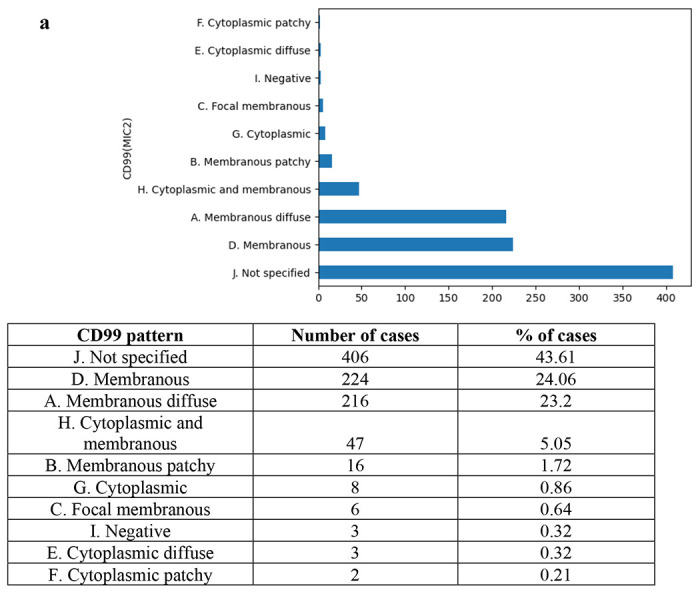

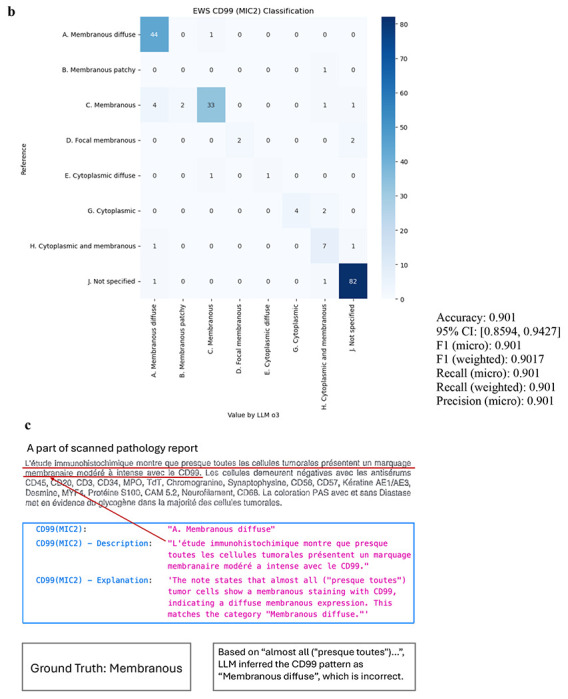
CD99 Pattern Identification

Beyond binary marker status, the LLM successfully categorized complex CD99 staining patterns described in the narrative text. Figure 3a illustrates the distribution of identified patterns. The most frequently specified patterns were “Membranous” (24.06%) and “Membranous Diffuse” (23.2%), while pure cytoplasmic staining was rare (1.4%). The model achieved an estimated accuracy of 90.1% (95% CI: 0.859–0.942) for pattern recognition. The confusion matrix for this task is presented in Figure 3b. Error analysis revealed that the primary source of classification error was distinguishing between “Membranous” and “Membranous Diffuse” categories based on ambiguous descriptions of tumor cell distribution, as detailed in Figure 3c. This example also highlights the opportunity to extract information from non-English narrative reports with LLMs’ multilingual capability.

### Prognostic Significance of Neuron-Specific Enolase (NSE).

**Figure 4.**
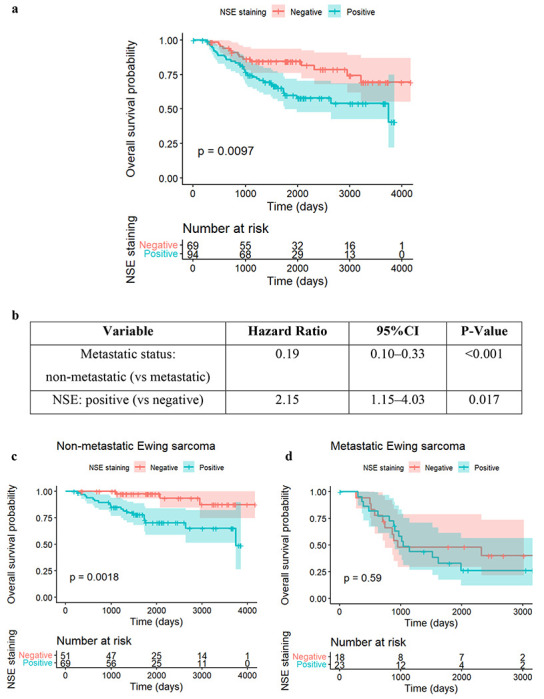

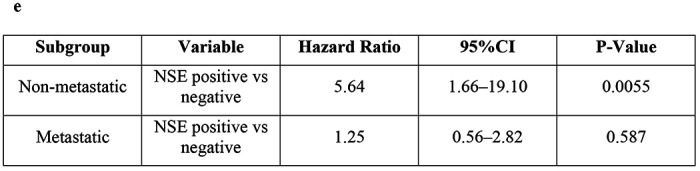
Survival analysis for IHC markers NSE **a.** Kaplan-Meier survival curves of NSE: the red curve represents the estimated probability of survival for patients with NSE negative, changing with time (unit: day); the shadow of the curve is the confidence interval; similarly, the blue curve represents the estimated probability of survival for patients with NSE positive, changing with time; this estimation with Kaplan-Meier model has p-value of 0.0097, thus being statistically significant. The “Number at risk” table below the curves shows the total numbers of patients with NSE negative or positive at different time points – at day 0, 69 patients with NSE negative and 94 patients with NSE positive are under observation; at day 1000, 55 patients with NSE negative and 68 patients with NSE positive are under observation, where the differences, 14 (69-55) patients either died or censored (stopping tracing for certain reasons); at day 4000, only 1 patient with NSE positive is still under observation. The difference between Kaplan-Meier survival curves for NSE positive and NSE negative illustrates the different survival probabilities, and patients with NSE positive have smaller probabilities to survive. **b.** After adjusting for metastatic versus non-metastatic disease at diagnosis, NSE positivity was associated with significantly worse survival. The hazard ratio for NSE-positive tumors was HR = 2.15 (p = 0.0166), indicating more than a two-fold higher risk of death compared to NSE-negative patients. **c.** Subgroup analysis for NSE in Non-Metastatic group. **d.** Subgroup analysis for NSE in Metastatic group. **e.** Subgroup analyses revealed that the prognostic effect of NSE was confined to patients with non-metastatic disease. Among non-metastatic patients, NSE positivity was associated with markedly worse survival (HR = 5.64, p = 0.0055), representing a very strong adverse prognostic signal. In contrast, among metastatic patients, NSE showed no association with survival (HR = 1.25, p = 0.587). This pattern suggests a possible biological interaction where NSE positivity distinguishes more aggressive tumors specifically within the localized disease group.

Survival analysis identified NSE as a significant prognostic factor. In the overall cohort, Kaplan-Meier analysis (Figure 4a) demonstrated that patients with NSE-positive tumors had significantly inferior overall survival (OS) compared to those with NSE-negative tumors (log-rank p = 0.0097).

We further assessed the independence of this marker using multivariable Cox proportional hazards regression. As summarized in Figure 4b, after adjusting for metastatic status, NSE positivity remained independently associated with worse survival (HR = 2.15, p = 0.016), indicating a more than two-fold increase in the risk of death compared to NSE-negative patients.

To determine if this risk was uniform across disease stages, we performed subgroup analyses. Among patients with non-metastatic disease (Figure 4c), NSE positivity was associated with markedly worse survival (HR = 5.64, p = 0.0055). In contrast, among patients with metastatic disease (Figure 4d), NSE showed no significant association with survival (HR = 1.25, p = 0.587). The summary of this interaction is presented in Figure 4e, suggesting that NSE positivity identifies a specifically high-risk subset of patients within the localized disease population who may otherwise be stratified as standard risk. Supplementary Table S2 presents the clinical features of patients with positive or negative NSE. S2a presents categorical variables and S2b presents numeric variables.

### Prognostic Significance of S100.

**Figure 5.**
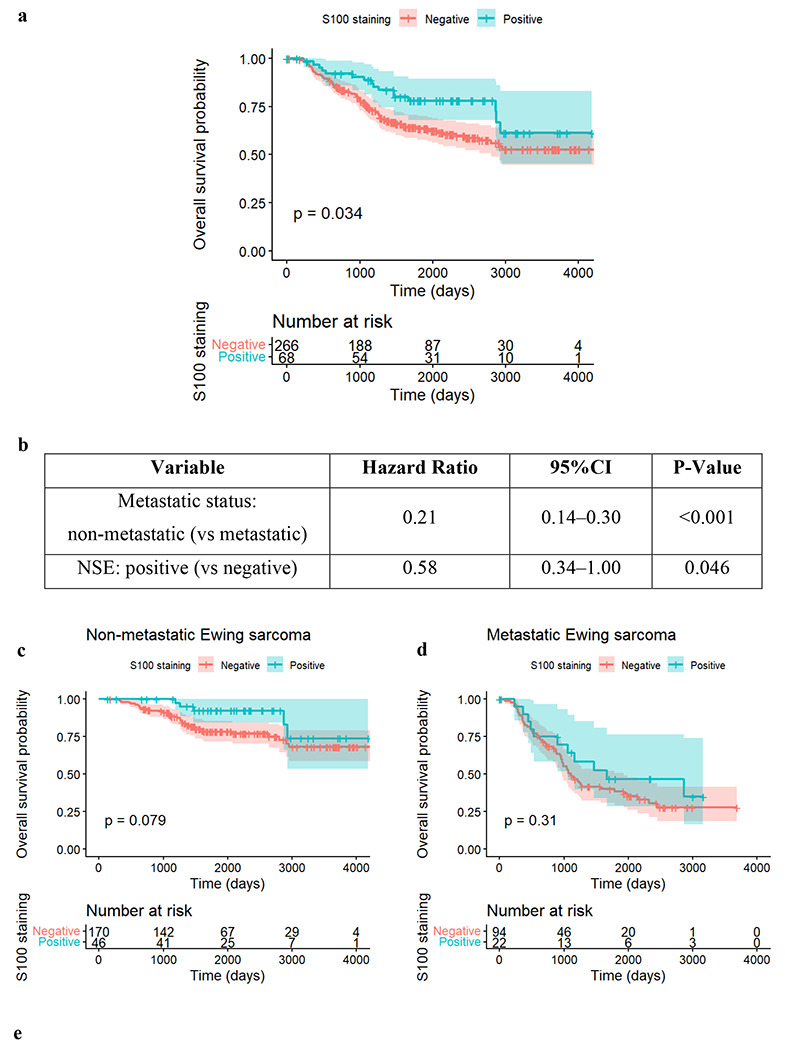

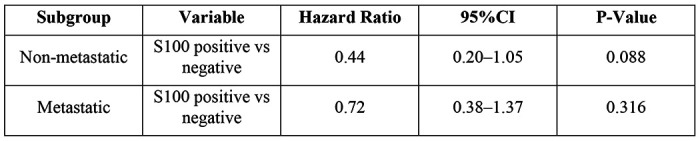
Survival analysis for IHC markers S100 **a.** The Kaplan-Meier analysis shows that patients with S100-positive tumors have better overall survival compared with those who are S100-negative. The log-rank test demonstrates a statistically significant difference (p < 0.034). These results indicate that S100 positivity is associated with improved overall survival in Ewing sarcoma, in contrast to the adverse prognostic effect observed with NSE.**b.** This table summarizes the results of a multivariable Cox regression model evaluating the association between S100 positivity and overall survival while adjusting for metastatic status at diagnosis. After accounting for metastatic status, S100-positive tumors demonstrated significantly better overall survival compared with S100-negative tumors (HR = 0.58, 95% CI: 0.34-1.00, p = 0.046). These findings support a protective effect of S100 expression in Ewing sarcoma.**c.** Subgroup analysis for S100 in Non-Metastatic group. **d**. Subgroup analysis for S100 in Metastatic group. **e**. This table presents subgroup survival analyses stratified by metastatic status. Among patients with non-metastatic disease, S100 positivity was associated with a lower hazard of death (HR = 0.44, 95% CI: 0.20–1.05), showing a trend toward improved survival, though this did not reach statistical significance (p = 0.088). S100 staining demonstrated no significant prognostic effect in the metastatic subgroup (HR = 0.72, 95% CI: 0.38–1.37, p = 0.316).

In contrast to the adverse risk associated with NSE, S100 expression was associated with improved outcomes. Kaplan-Meier analysis of the overall cohort (Figure 5a) showed that patients with S100-positive tumors had significantly better overall survival compared to those who were S100-negative (log-rank p = 0.034).

This protective effect persisted in multivariable analysis. Figure 5b details the Cox regression results. After adjusting for metastatic status, S100 positivity remained associated with improved survival (HR = 0.58, 95% CI: 0.34–1.00, p = 0.046). Figure 5c and 5d show subgroup survival curves of S100 adjusting for metastatic status in non-metastatic and metastatic groups respectively. Subgroup analyses revealed that, similar to NSE, the prognostic signal was most pronounced in the non-metastatic setting (HR = 0.44, p = 0.088), though it did not reach strict statistical significance in the subgroup. No significant effect was observed in the metastatic subgroup (HR = 0.72, p = 0.316), as summarized in Figure 5e. Supplementary Table S3 presents the clinical features of patients with positive or negative S100. S3a presents categorical variables and S3b presents numeric variables.

## Discussion

This article presents an efficient approach to leveraging LLMs to unlock information from historical clinical notes accumulated in COG for the research of Ewing sarcoma, a rare disease of young people.

Large language models have the potential to uncover patterns in medical documents, previously unrecognized. We applied an LLM to diagnostic pathology reports collected from 931 pediatric patients with Ewing sarcoma, representing hundreds of different institutions and expert pathologists, that were collected as scanned portable document format (PDF) documents secured from multiple clinical trials over 19 years. We implemented a data extraction procedure for 17 immunohistochemical stains that were performed in the diagnostic evaluation of each tumor biopsy according to each institution guidelines and individual pathologist direction. We also extracted 10 different descriptive varieties of CD99 staining pattern (including negative and not-specified). Neuron-specific enolase (NSE) and S100 staining are strongly predictive of patient outcome. LLMs successfully extract the structured information of IHC staining (with average accuracy of 94%) and CD99 patterns (with average accuracy 90.1%), from low quality error-prone scanned texts of EWS pathology reports. Structured data extracted by LLMs allows downstream data science for Ewing sarcoma, such as image classification by staining and potentially image selection for expert pathology annotation by staining pattern. Additionally extracted data for IHC markers together with the available structured clinical data (including overall survival time and life status), allowed survival analysis and found that NSE and S100 positivity are associated with overall survival in Ewing sarcoma. Overall, LLM technology is accurate, scalable and capable of revealing prognostic information previously unrecognized in Ewing sarcoma.

Data describing the application of LLMs to medical reports are emerging. Grothey and colleagues applied open source LLMs to bilingual pathology reports from a single institution and reported accuracy similar to proprietary models^19^. Danhauser and colleagues applied LLMs to fictious pediatric clinical reports and also demonstrated high accuracy in identifying patient information^20^. Balasubramanian and colleagues applying both proprietary and open-source LLMs to breast cancer pathology reports available as scanned images in tagged image file format (TIFF) from a Breast Cancer Now Generations study demonstrated accuracy comparable to human data extraction^12^. Each of these reports and others highlight the accuracy and scalability of LLMs applied to medical reports. Our data extends this further by first applying OCR technology to variable quality scanned PDF images retaining 96% consistency to human annotation over 17 individual immunohistochemical stains.

The College of American Pathologists (CAP) recommends the use of immunohistochemical stains in the evaluation of a presumed Ewing sarcoma diagnostic biopsy^21^. CD99 membranous staining (identified in 91% of cases) is considered essential for the diagnosis of Ewing sarcoma, while immunostains such as S100 (identified in 36% of cases), myogenin (identified in 52% of cases) and desmin (identified in 63% of cases) are useful adjuncts to rule out other small round cell tumors. NSE immunostaining is not recommended by CAP in the diagnostic evaluation of Ewing sarcoma, although 18% of patient biopsies were identified as stained in this data set. One prior report of 40-cases, suggested a possible link between NSE staining and patient outcome in non-metastatic Ewing sarcoma^22^, while the majority of prior reports find no association with outcome or do not study the relationship between immunohistochemical stain and patient outcome^23,24^. While previous data have suggested that neural differentiation may be associated with inferior outcomes, this appeared mitigated with more modern treatment approaches^25^. The apparent favorable outcome for patients with S100 staining, may be consistent with emerging data for patients with neurocristic tumors that like Ewing sarcoma are also FET::ETS fusion positive and merits further consideration ^26,27^. CD99 is associated with Ewing pathogenesis^28^, and while essential for diagnosis no reports link staining to outcome. We also found no association between CD99 and outcome. However, since molecular confirmation of Ewing sarcoma was not required for patients enrolled on the clinical trials in this dataset, different CD-99 staining pattern may suggest non-ETS round cell sarcoma which can be explored in future studies. Our finding that immunostaining in Ewing sarcoma is prognostic, challenges modern concepts in this rare tumor. We cannot tell why some institutional pathologists employed either NSE or S100 within their battery of immunostains, nor if these were added after an initial panel of stains for additional clarity. However, the observation that immunostaining features retain significant prognostic value when controlling for metastatic status strengthens the suggestive evidence.

Automated data extraction models require human annotation to create a ground truth from which to train the model. We address this obstacle by both annotating and cross validating immunohistochemical staining results by two clinicians on our study group. Application of artificial intelligent tools to rare disease prompts the risk of overfitting data in small single institution data series. We overcome this risk by compiling the largest series including clinical data and scanned pathology reports. Another risk of single data series is the ability to generalize findings to other institutional data. We overcome this risk by studying scanned pathology reports, generated by hundreds of pathologists, drawn from many institutions and institutions over 19 years. All these cases were considered eligible to be enrolled on COG clinical trials for Ewing sarcoma with diagnostic criteria acceptable at the time of the study. The fact that molecularly confirmation of Ewing sarcoma was not required for clinical trial eligibility adds an additional limitation to these data.

LLM technology demonstrates accuracy, scalability, and the ability to uncover previously unrecognized prognostic information in Ewing sarcoma. AI-derived histologic data show promise for refining risk stratification and should be validated in prospective clinical trials.

## Supplementary Material

1

## Figures and Tables

**Table 1.** Baseline Characteristics of the Ewing Sarcoma Cohort (N = 932)

Age at enrollment (years) Median (Q1, Q3);	13.5 (9.5, 16.6)
Sex	
Female	420 (45%)
Male	511 (55%)
Unknown	1 (0.1%)
Race	
American Indian or Alaska	6 (0.6%)
Asian	22 (2.4%)
Black or African American	22 (2.4%)
Multiple Races	6 (0.6%)
Native Hawaiian or other Pacific Islander	11 (1.2%)
Not Reported	14 (1.5%)
Unknown	72 (7.7%)
White	779 (84%)
Ethnicity	
Hispanic or Latino	129 (14%)
Not Hispanic or Latino	758 (81%)
Not reported	6 (0.6%)
Unknown	39 (4.2%)
Metastatic status at diagnosis	
Metastatic	328 (35%)
Non-metastatic	597 (64%)
Unknown	7 (0.8%)
Primary tumor site (grouped)	
Axial skeleton	277 (30%)
Long bones	226 (24%)
Other	288 (31%)
Soft tissue	94 (10%)
Unknown	47 (5.0%)
Follow-up time (days) Median (Q1, Q3);	1,602 (965, 2,477)
Life status	
Alive	657 (70%)
Dead	270 (29%)
Unknown	5 (0.5%)
*1* Median (Q1, Q3); n (%)	

**Table 2.** Evaluation of IHC data extraction performance (197)

IHC Marker	Cases	Accuracy	95% Confidence interval	F1 (weighted)	Recall (weighted)	Precision (weighted)
BCOR	2	1.000	[1.000, 1.000]	1.000	1.000	1.000
CD99(MIC2)	182	0.951	[0.918, 0.984]	0.971	0.951	0.994
CHROMOGRANIN	42	0.905	[0.810, 0.976]	0.942	0.905	0.988
DESMIN	128	0.945	[0.898, 0.984]	0.972	0.945	1.000
DUX4	1	1.000	[1.000, 1.000]	1.000	1.000	1.000
ERG	14	0.929	[0.786, 1.000]	0.962	0.929	1.000
ETV4	1	1.000	[1.000, 1.000]	1.000	1.000	1.000
FLI-1	58	0.914	[0.845, 0.983]	0.955	0.914	1.000
LCA(CD45)	131	0.962	[0.931, 0.992]	0.981	0.962	1.000
MyoD1	38	0.868	[0.763, 0.974]	0.916	0.868	0.971
Myogenin	101	0.931	[0.871, 0.970]	0.964	0.931	1.000
NKX2.2	23	0.913	[0.783, 1.000]	0.955	0.913	1.000
NSE	40	0.975	[0.925, 1.000]	0.987	0.975	1.000
S100	80	0.963	[0.913, 1.000]	0.981	0.963	1.000
SYNAPTOPHYSIN	85	0.941	[0.894, 0.988]	0.970	0.941	1.000
VIMENTIN	42	0.952	[0.881, 1.000]	0.972	0.952	1.000
WT1	45	0.889	[0.778, 0.978]	0.931	0.889	0.982
Weighted Average		0.940		0.966	0.939	0.997

**Table 3.** IHC ground truth cross validation

IHC marker	Consistency rate	95% Confidence interval (lb,ub)	
BCOR	0.979	0.938	1.000
CCNB3	1.000	1.000	1.000
CD99(MIC2)	0.958	0.896	1.000
CHROMOGRANIN	0.938	0.854	1.000
DESMIN	0.938	0.875	1.000
DUX4	0.979	0.938	1.000
ERG	1.000	1.000	1.000
ETV4	1.000	1.000	1.000
FLI-1	1.000	1.000	1.000
LCA(CD45)	0.854	0.749	0.938
MyoD1	0.979	0.938	1.000
Myogenin	0.958	0.896	1.000
NKX2.2	1.000	1.000	1.000
NSE	0.917	0.833	0.979
S100	0.938	0.854	1.000
SYNAPTOPHYSIN	0.958	0.896	1.000
VIMENTIN	0.958	0.896	1.000
WT1	0.979	0.938	1.000
Average	0.963		

**Table 4.** Evaluation of IHC labeling with consensus ground truth

IHC Variable	Number of cases	Accuracy (AB)	Accuracy (PL)	Accuracy (LLM: o3)
CD99(MIC2)	46	0.957	1.000	0.935
CHROMOGRANIN	12	0.833	0.917	1.000
DESMIN	35	0.943	0.971	0.971
DUX4	1	1.000	0.000	1.000
ERG	2	1.000	1.000	1.000
FLI-1	11	1.000	1.000	1.000
LCA(CD45)	35	0.829	0.914	1.000
MyoD1	12	0.917	0.833	1.000
Myogenin	28	0.929	1.000	1.000
NKX2.2	5	1.000	1.000	0.800
NSE	12	0.750	1.000	1.000
S100	18	0.889	0.944	1.000
SYNAPTOPHYSIN	26	0.962	0.962	1.000
VIMENTIN	10	0.800	1.000	1.000
WT1	13	1.000	0.923	1.000
Weighted Average		0.914	0.959	0.981

**Table 5.** Summary of CD99 Patterns

CD99 pattern	Number of cases	% of cases
J. Not specified	406	43.61
D. Membranous	224	24.06
A. Membranous diffuse	216	23.2
H. Cytoplasmic and membranous	47	5.05
B. Membranous patchy	16	1.72
G. Cytoplasmic	8	0.86
C. Focal membranous	6	0.64
I. Negative	3	0.32
E. Cytoplasmic diffuse	3	0.32
F. Cytoplasmic patchy	2	0.21
Total	931	100%

## Research in Context

### Evidence before this study

We searched PubMed and Google Scholar for articles published up to December 2025, using terms such as “Ewing sarcoma”, “prognosis”, “risk stratification”, “large language models”, “natural language processing”, and “pathology report extraction”. Current risk stratification for Ewing sarcoma relies predominantly on clinical variables, specifically the presence of metastatic disease and tumor size. While histologic heterogeneity is well-documented, it is rarely incorporated into risk models because extracting structured data from narrative pathology reports is labor-intensive. Small, single-institution studies have suggested potential prognostic roles for markers like Neuron-Specific Enolase (NSE) or S100, but results have been inconsistent and limited by small sample sizes. Furthermore, while Large Language Models (LLMs) have demonstrated potential for extracting clinical data, their application to “rescue” data from legacy, multi-institutional clinical trial documents (scanned PDF images) for rare disease biomarker discovery has not been validated at scale.

### Added value of this study

To our knowledge, this is the largest study to utilize AI to extract histologic data from Ewing sarcoma pathology reports, aggregating 931 patients from six distinct Children’s Oncology Group (COG) clinical trials spanning over 19 years. We validated an LLM-based pipeline that converted noisy, optically character-recognized text from diverse institutions into structured data with 98.1% accuracy, outperforming human annotators. This scalable approach revealed that NSE positivity is a significant independent risk factor for mortality (HR=2.15), particularly in patients with non-metastatic disease where it confers a more than 5-fold increased risk of death. Conversely, we identified S100 positivity as a protective factor associated with improved survival (HR=0.58).

### Implications of all the available evidence

This study demonstrates that LLMs can reliably unlock “dark data” from historical clinical trials, rendering vast archives of unstructured medical documents accessible for retrospective analysis. The identification of NSE and S100 as robust prognostic biomarkers suggests that these widely available immunohistochemical stains provide valuable information beyond standard diagnostic information. These findings support the integration of automated data extraction tools in research workflows and suggest that NSE and S100 status should be considered in the design of future risk-stratified clinical trials for Ewing sarcoma.
