# Supplementary material for: Leveraging Large Language Models to Extract Prognostic Pathology Features in Ewing Sarcoma
Source: bioRxiv. 2026 Feb 21:2026.02.20.707103. Preprint. [Version 1] doi: 10.64898/2026.02.20.707103 (PMC12934661; doi:10.64898/2026.02.20.707103)
Supplement: Supplementary file 1 [file NIHPP2026.02.20.707103v1-supplement-1.pdf]

bioRxiv preprint doi: https://doi.org/10.64898/2026.02.20.707103; this version posted February 22, 2026. The copyright holder for this preprint (which was not certified by peer review) is the author/funder, who has granted bioRxiv a license to display the preprint in perpetuity. It is made available under aCC-BY 4.0 International license.

Supplementary Materials

Table S1. IHC data extracted by LLM (930)

IHC variable	Positive	Negative	Not specified (NS)	% of Posi.	% of Neg.	% of NS
BCOR	3	7	920	0.32	0.75	98.61
CD99(MIC2)	846	4	80	90.87	0.43	85.9
CHROMOGRANIN	6	171	753	0.64	18.33	80.71
DESMIN	8	576	346	0.86	61.74	37.08
DUX4	0	5	925	0	0.54	99.14
ERG	17	20	893	1.82	2.14	95.71
ETV4	0	1	929	0	0.11	99.57
FLI-1	200	19	711	21.44	2.04	76.21
LCA(CD45)	5	574	351	0.54	61.52	37.62
MyoD1	5	119	806	0.54	12.75	86.39
Myogenin	2	477	451	0.21	51.13	48.34
NKX2.2	96	1	833	10.29	0.11	89.28
NSE	95	70	765	10.18	7.5	81.99
S100	68	266	596	7.29	28.51	63.88
SYNAPTOPHYSIN	154	227	549	16.51	24.33	58.84
VIMENTIN	192	13	725	20.58	1.39	77.71
WT1	9	182	739	0.96	19.51	79.21

Figure S1. Prompt for IHC data extraction

```
f"""You are an AI Assistant that follows instructions extremely well. You work as a pathologist assistant helping to extract immunohistochemistry stains from clinic notes in the field of Ewing Sarcoma.


# **Instructions**

Review the **Clinical Note**, provided in the end. From the clinical note, extract the following list of immunohistochemistry (IHC) variables:

**IHC variable list**
{{<Variable List>}}


For each IHC variable, assess the IHC stain regarding positive or negative, as it was stated in the **Clinical Note**, and select one of the three possible values as follows.

* A. Positive
* B. Negative
* C. Not specified


Note that the **Clinical Note** was scanned from paper documents, thus having common issues of raw scanned data, such as missing characters or misspelling. Have those possible scan errors "fixed" in mind if you can, when assessing each IHC variables.

Do not infer IHC stain, just based on what stated in the **Clinical Note**.


Output all of your answers to all IHC variables together in a flatten JSON data structure, as follows.

{{<json_output_template>}}

Don't output anything else beyond the above JSON data.


# **Clinical Note**
<Clinical Note>
{{<input_data>}}
</Clinical Note>
"""
```

Prompt template for IHC data extraction. In the template, {{<Variable List>}} will replaced with CD99(MIC2), NKX2.2, FLI-1, ERG, Myogenin, MyoD1, DUX4, WT1, ETV4, BCOR, LCA(CD45), SYNAPTOPHYSIN, CHROMOGRANIN, DESMIN, and VIMENTIN. {{<json_output_template>}}

bioRxiv preprint doi: https://doi.org/10.64898/2026.02.20.707103; this version posted February 22, 2026. The copyright holder for this preprint (which was not certified by peer review) is the author/funder, who has granted bioRxiv a license to display the preprint in perpetuity. It is made available under aCC-BY 4.0 International license.

will be replaced with the JSON output template provided. {{<input_data>}} will be replaced with each input pathology report.

Figure S2. JSON output template for IHC data extraction

CD99(MIC2) "<your assessment: select one of values among A, B, or C, based on the **Clinical Note**>"
CD99(MIC2) - Description "<the selected piece(s) of **original text** extracted from the **Clinical Note**, supporting your assessment>"
CD99(MIC2) - Explanation "<Explanation of your assessment of IHC variable CD99(MIC2)>"
CD99(MIC2) - Belief Degree "<A real number between 0 and 1, indicating your degree of belief in your assessment.>"
NKX2.2 "<select one of options among A, B, or C, based on the **Clinical Note**>"
NKX2.2 - Description "<the selected piece(s) of **original text** extracted from the **Clinical Note**, supporting your assessment>"
NKX2.2 - Explanation "<Explanation of your assessment of IHC variable NKX2.2>"
NKX2.2 - Belief Degree "<A real number between 0 and 1, indicating your degree of belief in your assessment.>"
FLI-1 "<select one of options among A, B, or C, based on the **Clinical Note**>"
FLI-1 - Description "<the selected piece(s) of **original text** extracted from the **Clinical Note**, supporting your assessment>"
FLI-1 - Explanation "<Explanation of your assessment of IHC variable FLI-1>"
FLI-1 - Belief Degree "<A real number between 0 and 1, indicating your degree of belief in your assessment.>"
ERG "<select one of options among A, B, or C, based on the **Clinical Note**>"
ERG - Description "<the selected piece(s) of **original text** extracted from the **Clinical Note**, supporting your assessment>"
ERG - Explanation "<Explanation of your assessment of IHC variable ERG>"
ERG - Belief Degree "<A real number between 0 and 1, indicating your degree of belief in your assessment.>"
Myogenin "<select one of options among A, B, or C, based on the **Clinical Note**>"
Myogenin - Description "<the selected piece(s) of **original text** extracted from the **Clinical Note**, supporting your assessment>"
Myogenin - Explanation "<Explanation of your assessment of IHC variable Myogenin>"
Myogenin - Belief Degree "<A real number between 0 and 1, indicating your degree of belief in your assessment.>"
MyoD1 "<select one of options among A, B, or C, based on the **Clinical Note**>"
MyoD1 - Description "<the selected piece(s) of **original text** extracted from the **Clinical Note**, supporting your assessment>"
MyoD1 - Explanation "<Explanation of your assessment of IHC variable MyoD1>"
MyoD1 - Belief Degree "<A real number between 0 and 1, indicating your degree of belief in your assessment.>"
DUX4 "<select one of options among A, B, or C, based on the **Clinical Note**>"
DUX4 - Description "<the selected piece(s) of **original text** extracted from the **Clinical Note**, supporting your assessment>"
DUX4 - Explanation "<Explanation of your assessment of IHC variable DUX4>"
DUX4 - Belief Degree "<A real number between 0 and 1, indicating your degree of belief in your assessment.>"
WT1 "<select one of options among A, B, or C, based on the **Clinical Note**>"
WT1 - Description "<the selected piece(s) of **original text** extracted from the **Clinical Note**, supporting your assessment>"
WT1 - Explanation "<Explanation of your assessment of IHC variable WT1>"
WT1 - Belief Degree "<A real number between 0 and 1, indicating your degree of belief in your assessment.>"
ETV4 "<select one of options among A, B, or C, based on the **Clinical Note**>"
ETV4 - Description "<the selected piece(s) of **original text** extracted from the **Clinical Note**, supporting your assessment>"
ETV4 - Explanation "<Explanation of your assessment of IHC variable ETV4>"
ETV4 - Belief Degree "<A real number between 0 and 1, indicating your degree of belief in your assessment.>"
BCOR "<select one of options among A, B, or C, based on the **Clinical Note**>"
BCOR - Description "<the selected piece(s) of **original text** extracted from the **Clinical Note**, supporting your assessment>"
BCOR - Explanation "<Explanation of your assessment of IHC variable BCOR>"
BCOR - Belief Degree "<A real number between 0 and 1, indicating your degree of belief in your assessment.>"

bioRxiv preprint doi: https://doi.org/10.64898/2026.02.20.707103; this version posted February 22, 2026. The copyright holder for this preprint (which was not certified by peer review) is the author/funder, who has granted bioRxiv a license to display the preprint in perpetuity. It is made available under aCC-BY 4.0 International license.

Figure S3. Prompt for CD99 pattern identification

```
f"""You are an AI Assistant that follows instructions extremely well. You work as a pathologist assistant helping to extract requested attributes from clinic notes in the field of Ewing Sarcoma.


# **Instructions**

Review the **Clinical Note**, provided in the end. From the clinical note, extract the following attributes (variable-value pairs), which are specified as a Variable-Value table in the form of JSON data, where a variable is a key, and the value of the key is a list of possible values for the variable:

**Variable-Value Table**
<Variable-Value Table>
{{<Variable-Value Dictionary>}}
</Variable-Value Table>


Note that the **Clinical Note** was scanned from paper documents, thus having common issues of raw scanned data, such as missing characters or misspelling. Have those possible scan errors "fixed" in mind if you can, when assessing each attribute.

Please make sure to extract the values based on what stated in the **Clinical Note**, and **do not infer** the values.


Output all of your answers to all requested attributes together in a flatten JSON data structure, as follows.

{{<json_output_template>}}

Don't output anything else beyond the above JSON data.


# **Clinical Note**
<Clinical Note>
{{<input_data>}}
</Clinical Note>
"""
```

```
{
    "CD99(MIC2)": [
        "A. Membranous diffuse",
        "B. Membranous patchy",
        "C. Focal membranous",
        "D. Membranous",
        "E. Cytoplasmic diffuse",
        "F. Cytoplasmic patchy",
        "G. Cytoplasmic",
        "H. Cytoplasmic and membranous",
        "I. Negative",
        "J. Not specified"
    ]
}
```

```
{
    "CD99(MIC2)": "<your assessment: based on the **Clinical Note**, select one of the standard values defined in Variable-Value Table>",
    "CD99(MIC2) - Description": "<the selected piece(s) of **original text** extracted from the **Clinical Note**, supporting your assessment>",
    "CD99(MIC2) - Explanation": "<explain your assessment>"
}
```

bioRxiv preprint doi: https://doi.org/10.64898/2026.02.20.707103; this version posted February 22, 2026. The copyright holder for this preprint (which was not certified by peer review) is the author/funder, who has granted bioRxiv a license to display the preprint in perpetuity. It is made available under aCC-BY 4.0 International license.

Table S2. Clinical data distribution over NSE

a.

Variable	Value	Number of Positive NSE	Percentage of Positive NSE	Number of Negative NSE	Percentage of Negative NSE
Gender	Female	48	50.53	36	51.43
	Male	47	49.47	34	48.57
Race	White	80	84.21	56	80.00
	Unknown	9	9.47	8	11.43
	Black or African American	3	3.16	3	4.29
	Asian	1	1.05	2	2.86
	Not Reported	1	1.05	1	1.43
	Native Hawaiian or other Pacific Islander	1	1.05	0	0
Ethnicity	Not Hispanic or Latino	84	88.42	59	84.29
	Hispanic or Latino	9	9.47	9	12.86
	Unknown	2	2.11	2	2.86
Primary Tumor Site	Osseous	60	63.16	56	80.00
	Extraosseous	25	26.32	12	17.14
	Not specified	10	10.53	2	2.86
Metastatic Status at Diagnosis	Non-metastatic	69	72.63	51	72.86
	Metastatic	24	25.26	18	25.71
	NA	2	2.11	1	1.43
Life Status	Alive	58	61.05	55	78.57
	Dead	36	37.89	14	20.00
	NA	1	1.05	1	1.43

b.

Var Stat	Value with Positive NSE	Value with Negative NSE
Age at enrollment (Years)(n)	95	70
Median	13.24	12.24
Q1	8.88	7.56
Q3	15.85	16.06
PercentTumorVsNecrosis(n)	95	70
Median	20.00	23.00
Q1	12.00	14.38
Q3	36.00	37.75
PercentTumorVsStroma(n)	95	70
Median	20.00	23.00
Q1	12.00	14.38
Q3	36.00	37.75
Overall Survival Time (Days)(n)	95	70
Median	1519.00	1898.00
Q1	977.50	1109.00
Q3	2082.50	2949.00

bioRxiv preprint doi: https://doi.org/10.64898/2026.02.20.707103; this version posted February 22, 2026. The copyright holder for this preprint (which was not certified by peer review) is the author/funder, who has granted bioRxiv a license to display the preprint in perpetuity. It is made available under aCC-BY 4.0 International license.

Table S3. Clinical data distribution over S100

a.

Variable	Value	Number of Positive NSE	Percentage of Positive NSE	Number of Negative NSE	Percentage of Negative NSE
Gender	Female	38	55.88%	144	54.14%
	Male	29	42.65%	122	45.86%
	NA	1	1.47%		
Race	White	58	85.29%	219	82.33%
	Unknown	4	5.88%	26	9.77%
	Black or African American	2	2.94%	3	1.13%
	Asian	2	2.94%	1	0.38%
	Not Reported	1	1.47%	6	2.26%
	Native Hawaiian or other Pacific Islander	1	1.47%	5	1.88%
Ethnicity	Not Hispanic or Latino	58	85.29%	205	77.07%
	Hispanic or Latino	8	11.76%	49	18.42%
	Unknown	1	1.47%	11	4.14%
Primary Tumor Site	Osseous	1	1.47%	1	0.38%
	Extraosseous	37	54.41%	172	64.66%
	Not specified	24	35.29%	73	27.44%
Metastatic Status at Diagnosis	Non-metastatic	7	10.29%	21	7.89%
	Metastatic	46	67.65%	170	63.91%
	NA	22	32.35%	94	35.34%
Life Status	Alive	52	76.47%	169	63.53%
	Dead	16	23.53%	97	36.47%

b.

Var Stat	Value with Positive S100	Value with Negative S100
Age at enrollment (Years)(n)	68	266
Median	14.36	14.54
Q1	9.85	10.18
Q3	16.89	16.84
PercentTumorVsNecrosis(n)	68	266
Median	25.00	30.00
Q1	10.00	10.00
Q3	38.75	46.25
PercentTumorVsStroma(n)	68	266
Median	25.00	30.00
Q1	10.00	10.00
Q3	38.75	46.25
Overall Survival Time (Days)(n)	68	266
Median	1823.00	1459.50
Q1	1162.50	878.25
Q3	2580.00	2251.50

bioRxiv preprint doi: https://doi.org/10.64898/2026.02.20.707103; this version posted February 22, 2026. The copyright holder for this preprint (which was not certified by peer review) is the author/funder, who has granted bioRxiv a license to display the preprint in perpetuity. It is made available under aCC-BY 4.0 International license.
